# Satellite DNA in *Paphiopedilum* subgenus *Parvisepalum* as revealed by high-throughput sequencing and fluorescent in situ hybridization

**DOI:** 10.1186/s12864-018-4956-7

**Published:** 2018-08-02

**Authors:** Yung-I Lee, Jing Wei Yap, Shairul Izan, Ilia J. Leitch, Michael F. Fay, Yi-Ching Lee, Oriane Hidalgo, Steven Dodsworth, Marinus J. M. Smulders, Barbara Gravendeel, Andrew R. Leitch

**Affiliations:** 1 Biology Department, National Museum of Natural Science, No 1, Kuan-Chien Rd, 40453 Taichung, Taiwan, Republic of China; 2 Department of Life Sciences, National Chung Hsing University, 40227 Taichung, Taiwan, Republic of China; 3 School of Biological and Chemical Sciences, Queen Mary University of London, London, E1 4NS UK; 4 Jodrell Laboratory, Royal Botanic Gardens, Kew, Richmond, Surrey, TW9 3AB UK; 5 Forest Research Institute Malaysia (FRIM), 52109 Kepong, Selangor Darul Ehsan Malaysia; 6 Plant Breeding, Wageningen University & Research, P.O. Box 386, NL-6700 AJ Wageningen, The Netherlands; 7 Department of Crop Science, Faculty of Agriculture, University Putra Malaysia (UPM) Serdang, Serdang, Selangor Malaysia; 8 Department of Comparative Plant and Fungal Biology, Royal Botanic Gardens, Kew, Richmond, Surrey, TW9 3AB UK; 9 School of Plant Biology, University of Western Australia, Crawley, WA 6009 Australia; 10 Endless Forms Group, Naturalis Biodiversity Center, Vondellaan 55, 2332 AA Leiden, The Netherlands; 11 Faculty of Science and Technology, University of Applied Sciences Leiden, Zernikedreef 11, 2333 CK Leiden, The Netherlands; 12 Institute Biology Leiden, Leiden University, Sylviusweg 72, 2333 BE Leiden, The Netherlands

**Keywords:** *Paphiopedilum*, Karyotype, Satellite DNA, Fluorescent in situ hybridization, FISH

## Abstract

**Background:**

Satellite DNA is a rapidly diverging, largely repetitive DNA component of many eukaryotic genomes. Here we analyse the evolutionary dynamics of a satellite DNA repeat in the genomes of a group of Asian subtropical lady slipper orchids (*Paphiopedilum* subgenus *Parvisepalum* and representative species in the other subgenera/sections across the genus). A new satellite repeat in *Paphiopedilum* subgenus *Parvisepalum*, SatA, was identified and characterized using the RepeatExplorer pipeline in HiSeq Illumina reads from *P. armeniacum* (2n = 26). Reconstructed monomers were used to design a satellite-specific fluorescent in situ hybridization (FISH) probe. The data were also analysed within a phylogenetic framework built using the internal transcribed spacer (ITS) sequences of 45S nuclear ribosomal DNA.

**Results:**

SatA comprises c. 14.5% of the *P. armeniacum* genome and is specific to subgenus *Parvisepalum*. It is composed of four primary monomers that range from 230 to 359 bp and contains multiple inverted repeat regions with hairpin-loop motifs. A new karyotype of *P. vietnamense* (2n = 28) is presented and shows that the chromosome number in subgenus *Parvisepalum* is not conserved at 2n = 26, as previously reported. The physical locations of SatA sequences were visualised on the chromosomes of all seven *Paphiopedilum* species of subgenus *Parvisepalum* (2n = 26–28), together with the 5S and 45S rDNA loci using FISH. The SatA repeats were predominantly localisedin the centromeric, peri-centromeric and sub-telocentric chromosome regions, but the exact distribution pattern was species-specific.

**Conclusions:**

We conclude that the newly discovered, highly abundant and rapidly evolving satellite sequence SatA is specific to *Paphiopedilum* subgenus *Parvisepalum*. SatA and rDNA chromosomal distributions are characteristic of species, and comparisons between species reveal that the distribution patterns generate a strong phylogenetic signal. We also conclude that the ancestral chromosome number of subgenus *Parvisepalum* and indeed of all *Paphiopedilum* could be either 2n = 26 or 28, if *P. vietnamense* is sister to all species in the subgenus as suggested by the ITS data.

**Electronic supplementary material:**

The online version of this article (10.1186/s12864-018-4956-7) contains supplementary material, which is available to authorized users.

## Background

Nuclear genomes of higher plants are composed of coding and regulatory sequences and substantial amounts of repetitive DNA [including e.g. (retro) transposable elements and tandemly repeated DNA] that are considered to play important roles in genome differentiation, dynamics and evolution [1–4]. Repetitive DNA contributes to the diversity of genome sizes encountered in plants [5–7]. Satellite DNA (Sat) constitutes highly amplified tandemly repeated sequences, that can vary in abundance, sequence and chromosomal distribution between species [7–16]. Often it occurs in heterochromatic, peri-centromeric or sub-telomeric regions of the chromosome, but it can also be in found in interstitial regions. Because of the dynamic nature of the organization and distribution of these types of repeats, the characterization of satellite DNA is particularly useful for chromosome identification and for reconstruction of patterns of species divergence [13, 17–22].

*Paphiopedilum* (Orchidaceae: Cypripedioideae) is the most diverse genus of terrestrial slipper orchids, containing about 80 species, nearly all of which are rare and threatened [23]. The genus consists of seven subgroups, comprising three subgenera (*Parvisepalum*, *Brachypetalum* and *Paphiopedilum*) and five sections in subgenus *Paphiopedilum* (*Paphiopedilum*, *Cochlopetalum*, *Coryopedilum*, *Pardalopetalum* and *Barbata*) based on morphological, cytological and molecular phylogenetic data [24–28]. Patterns of speciation in some sections are complex and potentially involve recurrent patterns of interspecific hybridization, arising from the redistribution of taxa with changing sea levels across South East Asia during the glacial cycles of the late Cenozoic [28, 29].

The genus is characterized by considerable chromosome number variation (2n = 26–42) and a relatively wide range of genome sizes (2.2-fold, 1C = 16.5–35.9 pg, mean 1C = 25.4 pg) [27]. Previous cytological studies have suggested that Robertsonian translocations have contributed to the diversity of chromosome numbers observed between *Paphiopedilum* species, involving the fission of metacentric chromosomes at or near the centromere to generate telocentric chromosomes [30–37]. In addition, it is clear from more recent cytological studies that other types of complex chromosomal rearrangements (e.g. inversions and duplications) may also have contributed to the karyotypic diversity observed [38].

Little is known about the composition, diversity and evolutionary dynamics of repetitive DNA sequences in the genomes of *Paphiopedilum* species. In a study of ribosomal DNA (rDNA) sequence evolution by Lan and Albert [38], no clear relationships were uncovered between the number of rDNA signals, chromosome number and genome size. Duplications of the nuclear 45S rDNA locus occurred independently in subgenus *Parvisepalum* and sections *Coryopedilum* and *Pardalopetalum* of subgenus *Paphiopedilum*, whereas duplications of 5S rDNA loci were only observed in subgenus *Paphiopedilum*.

Recently, with developments in high-throughput sequencing approaches, non-model species have become more amenable to in-depth analyses of the repetitive DNA component of their genomes [7, 39, 40]. Even for species with large genomes (1C > 10 Gbp), it is possible to gain insights into the types, amounts, diversity and evolution of the most abundant repetitive elements using low-coverage sequencing data [14, 15, 41–47].

In this study, we use low-coverage genomic DNA sequence data from the Illumina next-generation sequencing platform to characterize the satellite DNA component of seven *Paphiopedilum* species, selected to represent the phylogenetic diversity of the genus. We undertook an in-depth analysis of the most abundant satellite DNA identified in the genus, which was identified in *P. armeniacum*. This species belongs to subgenus *Parvisepalum*, a lineage that is considered to have diverged from the rest of *Paphiopedilum* early in the evolution of the genus. In addition, we examined the chromosomal distribution of SatA in closely related species of subgenus *Parvisepalum* and representative species belonging to the other two subgenera, to provide a phylogenetic perspective of its distribution and evolution across the genus. Finally, we explored the utility of SatA as a chromosomal marker for characterizing karyotype evolution in species belonging to subgenus *Parvisepalum*.

## Results

### Phylogenetic relationships in *Paphiopedilum* subgenus *Parvisepalum*

Nuclear ribosomal ITS sequences were used to reconstruct phylogenetic relationships of the seven *Paphiopedilum* species belonging to subgenus *Parvisepalum* (Additional file 1: Fig. S1). The analysis re-confirmed the monophyly of the subgenus and resolved *P. vietnamense* as sister to the other species (bootstrap = 93%; PP = 1). In addition, two clades within the subgenus were recovered with strong to moderate support, the first consisting of *P. hangianum* and *P. emersonii* (bootstrap = 93%; PP = 1) and the other comprising *P. armeniacum*, and *P. malipoense*, (bootstrap = 69%; PP = 0.97). However, relationships between these clades and the two remaining species of the subgenus, i.e. *P. delenatii* and *P. micranthum,* remained unresolved.

### Satellite DNA identification and characterization

Using RepeatExplorer to individually cluster the Illumina HiSeq data for seven *Paphiopedilum* taxa (corresponding to between c. 0.84 and 7.6% of the genome depending on the taxon, Additional file 2: Table S1), we identified four distinct types of satellite DNA (SatA*,* SatB*,* SatG*,* and SatJ) based on the shape of the output graphs. The abundance of each repetitive DNA type varied between species (Table 1), with none containing all four satellites. The amount of satellite DNA was also estimated for the sister genus *Phragmipedium*, in which only SatG was identified, occurring in low abundance (0.04%) in the *P. longifolium* genome.

**Table 1:** Major types of repetitive DNA in *Paphiopedilum*

Subgenus/Section								
	Outgroup *Phragmipedium longifolium* %	Parvisepalum *P. armeniacum* %	Brachypetalum *P. concolor* %	Cochlopetalum *P. primulinum* %	Coryopedilum *P. rothschildianum* %	Pardalopetalum *P. lowii* %	Paphiopedilum *P. villosum* %	Barbata *P. appletonianum* %
Satellites								
*SatA*	0.00	14.39	0.00	0.00	0.00	0.00	0.00	0.00
*SatB*	0.00	0.10	0.13	6.18	2.81	7.45	1.07	4.11
*SatG*	0.04	0.00	0.00	0.46	0.65	1.63	0.69	0.42
*SatJ*	0.00	0.00	0.00	0.00	0.00	0.00	2.51	1.80
Total Satellites	0.04	14.49	0.13	6.64	3.47	9.08	4.27	6.33
LTR elements								
*Ty3/gypsy*								
*Ogre/Tat*	30.04	31.80	48.86	38.03	44.98	39.06	46.32	40.48
*Chromovirus*	7.82	2.49	1.47	0.59	1.44	0.81	0.89	0.66
*Athila*	0.20	4.41	1.21	0.21	1.19	0.18	0.28	0.14
Total Ty3/gypsy	38.06	38.70	51.54	38.83	47.61	40.05	47.49	41.28
*Ty1/copia*								
*Maximus/SIRE*	3.05	2.24	1.56	0.97	1.49	0.48	0.99	0.62
*Ivana*	0.00	3.14	2.14	1.14	1.92	0.81	2.74	1.82
*Tork*	7.17	0.00	0.16	0.05	0.04	0.08	0.05	0.07
Total Ty1/copia	10.22	5.38	3.86	2.16	3.45	1.37	3.78	2.51
Other repetitive elements								
TRIM	0.00	0.00	0.00	0.00	0.00	0.00	0.00	0.00
LINE	1.09	0.57	0.49	0.10	0.76	0.53	0.36	0.26
DNA transposon	0.60	2.41	1.97	1.31	2.02	1.25	1.63	1.83
MITE	0.00	0.00	0.00	0.00	0.00	0.00	0.00	0.00
rDNA	0.14	0.04	0.04	0.06	0.00	0.00	0.02	0.00
SSR	0.23	0.00	0.00	0.00	0.00	0.00	0.00	0.00
Pararetrovirus	0.00	0.08	0.00	0.00	0.16	0.00	0.00	0.59
Unclassified repetitive	17.31	9.86	10.60	11.94	10.36	9.90	10.63	11.38
Low and single copy	32.26	28.46	31.37	38.94	32.14	37.80	31.82	35.83
Total % repetitive DNA	61.18	62.04	59.54	50.43	59.26	53.62	58.64	53.88

SatA appeared to be specific to subgenus *Parvisepalum*, and it accounted for 14.4% of the *P. armeniacum* genome (Table 1). In contrast, SatB was found in all *Paphiopedilum* subgroups, ranging from 0.1% in *P. armeniacum* and *P. concolor* (subgenus *Parvisepalum* and section *Brachypetalum*, respectively) to 7.5% in *P. lowii* (section *Pardalopetalum*), although it was absent in the outgroup *P. longifolium*. SatG was found in both *Phragmipedium* and most *Paphiopedilum* species analysed with the exception of *P. armeniacum* (subgenus *Parvisepalum*) and *P. concolor* (subgenus *Brachypetalum*), in which it appears to have been lost or to be present in amounts below the threshold of detection used here. SatJ was found exclusively in just two sections of subgenus *Paphiopedilum*, comprising 2.5 and 1.8% of the *P. villosum* (section *Paphiopedilum*) and *P. appletonianum* (section *Barbata*) genomes, respectively (Table 1).

### Characterization of SatA in subgenus *Parvisepalum*

The characteristics of the top four most abundant SatA monomers in *P. armeniacum* are summarized in Additional file 3: Table S2 and Additional files 4, 5 and 6: Figs. S2-S4. All have high AT content (c. 66%) and contain multiple, often long stretches, of inverted repeat regions which form multiple hairpin loop motifs interspersed with unpaired bases (Additional file 4: Fig. S2). The largest (359 bp) monomer (CL1_965) is made up of three highly similar 146, 146 and 67 bp long repeat subunits (Additional file 6: Fig. S4) and is the most abundant high BLAST similarity hit in an all-to-all sequence comparison to nearly 50% of all SatA reads. The remaining three monomers (CL1_940, CL1_393 and CL1_886) are distinct from CL1_965. They ranged in length from 235 to 307 bp and possessed high sequence similarity (above 90%) to each other and as such can be aligned easily.

### Chromosomal organization of SatA and rDNA

#### SatA distribution patterns

**Fig. 1:**
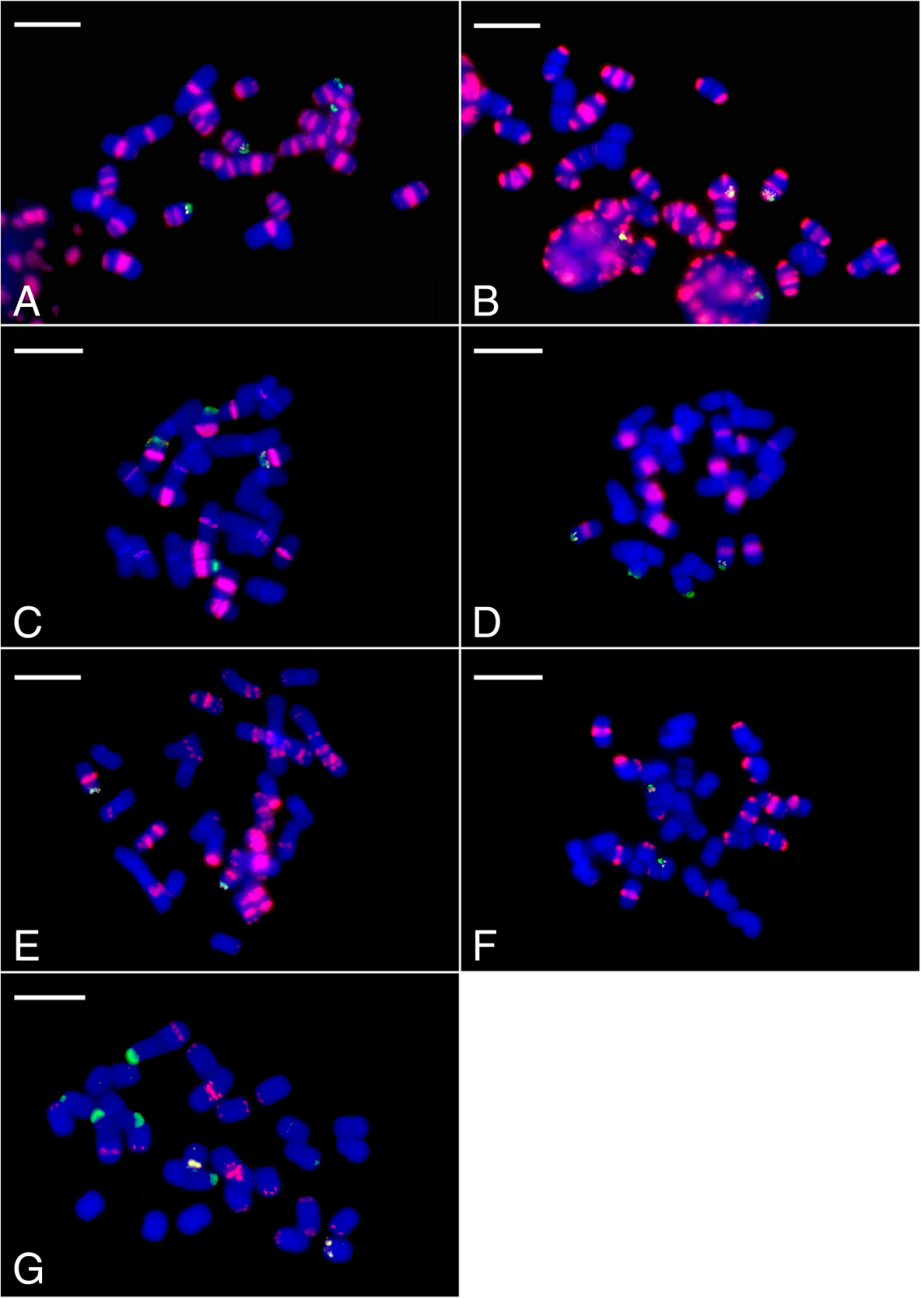
Fluorescent in situ hybridization (FISH) of root tip metaphase chromosomes of species belonging to subgenus *Parvisepalum* with the SatA (red), 45S (green) and 5S (white) rDNA probes, counterstained with DAPI (blue): (**a**) *P. armeniacum*, (**b**) *P. malipoense*, (**c**) *P. emersonii*, (**d**) *P. hangianum*, (**e**) *P. micranthum*, (**f**) *P. delenatii*, (**g**) *P. vietnamense*. Bar = 10 μm

**Fig. 2:**
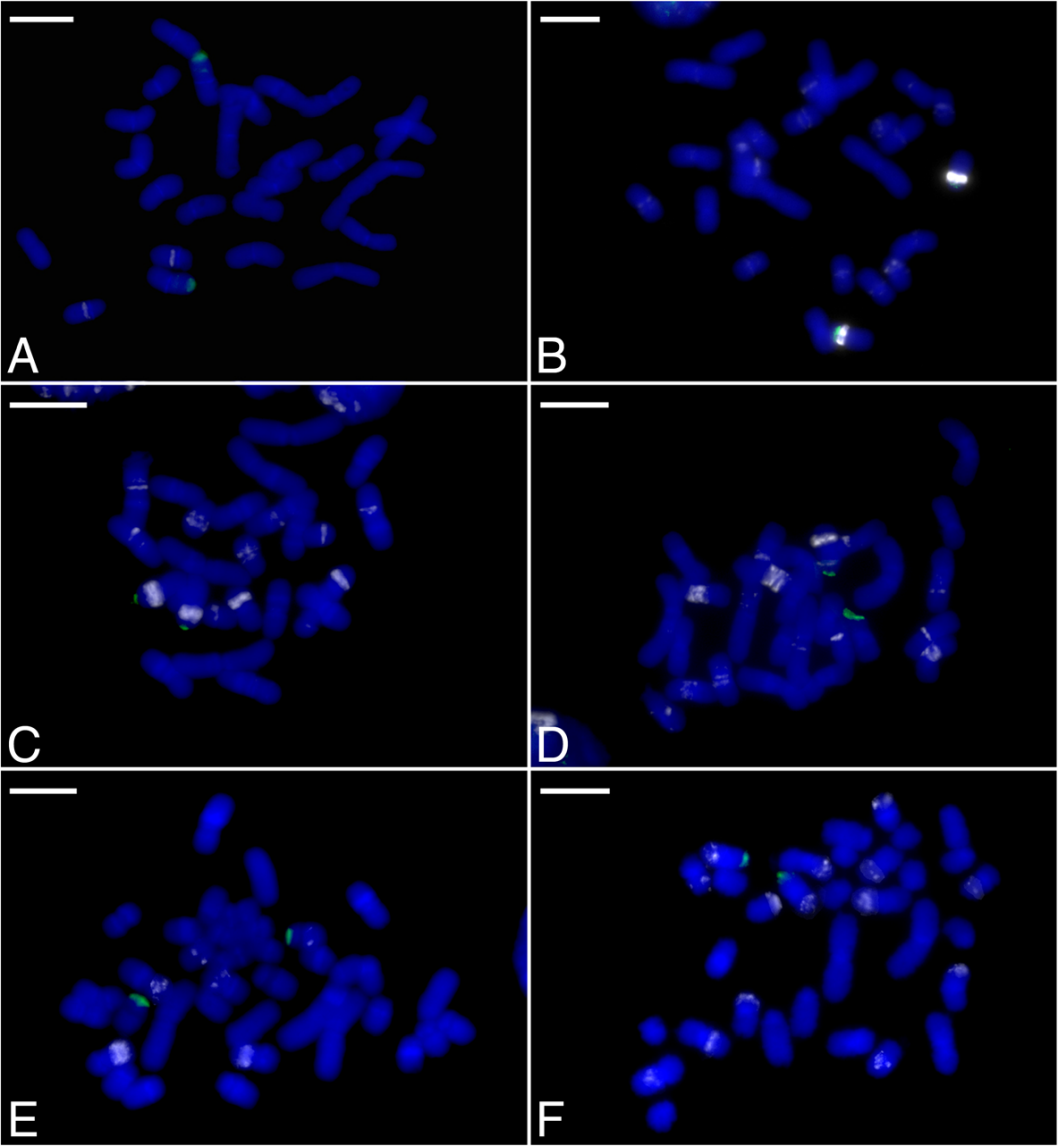
Fluorescent in situ hybridization (FISH) of root tip metaphase chromosomes with the SatA, 45S (green) and 5S (white) rDNA probes, counterstained with DAPI (blue): (**a**) *P. concolor* (subgenus *Brachypetalum*), (**b**) *P. villosum* (section *Paphiopedilum*), (**c**) *P. rothschildianum* (section *Coryopedilum*), (**d**) *P. lowii* (section *Pardalopetalum*), (**e**) *P. appletonianum* (section *Barbata*), and (**f**) *P. primulinum* (section *Cochlopetalum*). The absence of FISH signals confirms that SatA is indeed specific to subgenus *Parvisepalum*. Bar = 10 μm

The physical locations of long (> 1000 bp = the lower threshold of FISH sensitivity) [48] stretches of SatA sequence were visualized using FISH and showed that SatA hybridized to all species of subgenus *Parvisepalum* (Fig. 1). In contrast, none of the representative species belonging to the other subgenera or sections of *Paphiopedilum* had any hybridization signal. Both 45S and 5S rDNA probes hybridized to the same chromosome preparations (Fig. 2).

**Fig. 3:**
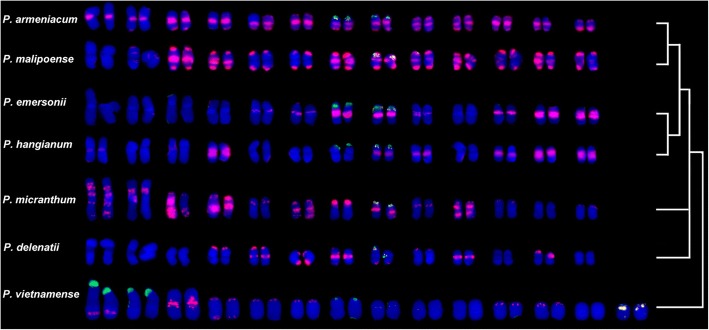
Fluorescent in situ hybridization of karyotypes of *Paphiopedilum* subgenus *Parvisepalum* with the SatA (red), 45S (green) and 5S (white) rDNA probes, counterstained with DAPI (blue). Phylogenetic relationships between these species shown on the right-hand side of the figure (see also Additional file 1: Fig. S1)

**Fig. 4:**
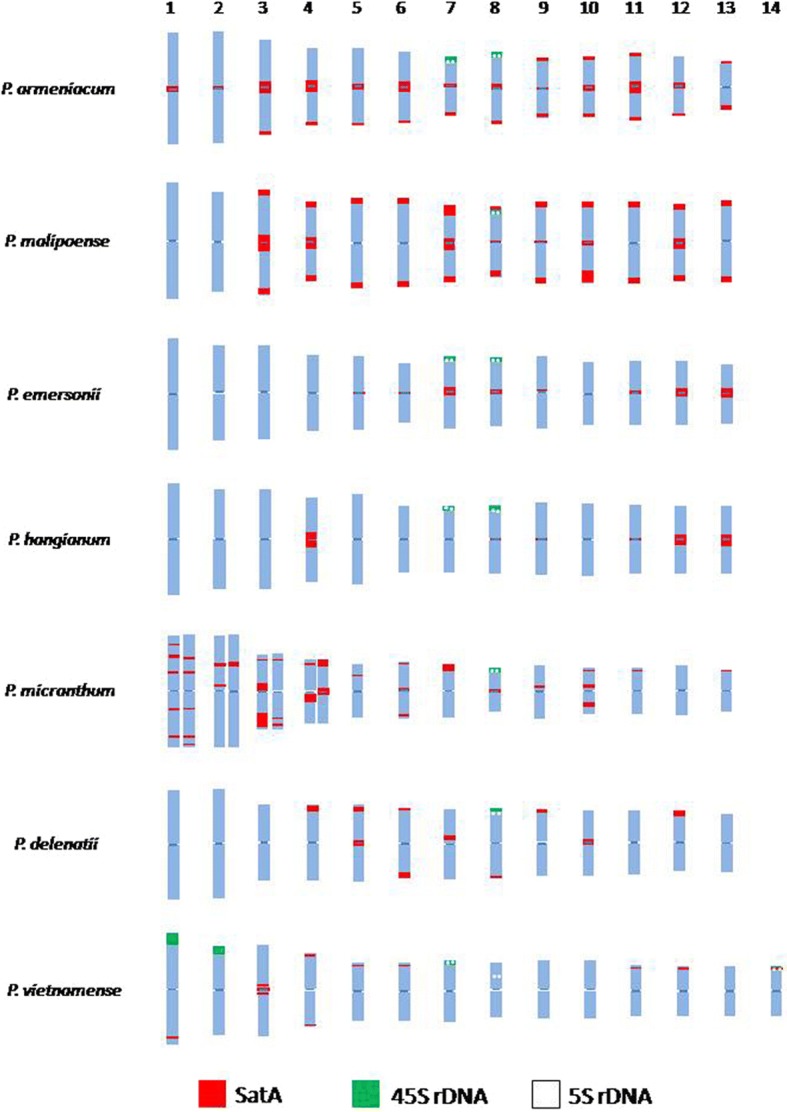
Ideograms of somatic metaphase chromosomes of species belonging to subgenus *Parvisepalum*.SatA (red), 45S (green) and 5S (white) rDNA signals

Species of *Paphiopedilum* subgenus *Parvisepalum* have 2n = 2× = 26 (except for *P. vietnamense*, 2n = 2× = 28), with mostly metacentric or sub-metacentric chromosomes. However, despite this apparent karyotypic uniformity, the hybridization pattern of the SatA probe was seen to differ considerably between species (Fig. 3). Thus to compare the physical distribution of the SatA hybridization sites in a phylogenetic context, karyotypes (Fig. 3) and ideograms (Fig. 4) of all seven species were prepared and arranged according to the nrITS phylogenetic tree. The arrangement of the chromosomes shown in these figures assumes that the SatA and rDNA signals are most likely carried on homologous chromosomes in the most closely related species, using approaches developed in Lim et al. [49] (Figs. 3 and 4).

The greatest abundance of SatA signals (which broadly reflects the genome proportion of 14.4% estimated by RepeatExplorer; see Table 1) was found in *P. armeniacum*, where all chromosomes had at least one site of SatA sequences. The signals were mainly located in the centromeric (defined here to include both the centromere and peri-centromeres) and sub-telomeric regions, although the precise position, strength and frequency of signals varied between chromosomes. Broadly similar patterns were also observed in *P. malipoense*. In contrast, clear SatA signals in *P. hangianum* and *P. emersonii* were only found in the centromeric regions of about half the chromosomes. For the remaining species analysed, both centromeric and sub-telomeric SatA signals were detected on at least some of the chromosomes, although the exact position, and strength of signal differed between species. Overall, the most abundant and intense centromeric and sub-telomeric SatA signals were observed in *P. armeniacum* and *P. malipoense*, whereas the weakest signals were found in *P. vietnamense* (Fig. 1).

By arranging karyotypes in a phylogenetic framework as predicted from nrITS sequence data (Fig. 3), the distribution of the SatA sequences is more similar in closely related species than more distantly related species, as illustrated by the following examples. (1) The distribution of the SatA sites in *P. armeniacum* were broadly similar to those in *P. malipoense* to which it is closely related. Nevertheless, although SatA sites were detected in centromeric and sub-telocentric regions of all eight chromosome pairs in *P. malipoense*, six chromosome pairs lacked prominent SatA signals in the centromeric region. These strong SatA signals coincided with heterochromatic bands seen by DAPI staining (data not shown) which became apparent after the denaturation step of the FISH protocol. (2) *P. emersonii* and *P. hangianum* are closely related sister species and both possessed SatA sites primarily in the peri-centromeric region. Nevertheless, in *P. hangianum*, the SatA signals co-localized exclusively with the heterochromatic bands in the centromeric region of six chromosome pairs, whereas in *P. emersonii*, SatA signals were restricted to the centromeric regions of just eight chromosome pairs, despite heterochromatic bands being present in the centromeric region of all chromosomes (data not shown).

In contrast to these species, *P. delenatii* showed only weak SatA signals in the peri-centromeric, sub-telocentric and interstitial regions. The distribution of SatA and rDNA signals in *P. vietnamense* is highly distinctive in comparison with the other species. In *P. micranthum,* multiple strongly dispersed interstitial signals across several chromosomes were observed. In addition, several hemizygous sub-telomeric *Sat*A signals, including one which spanned almost the entire length of one chromosome, were found in *P. micranthum* (Figs. 1e, 3 and 4).

#### rDNA distribution patterns

The 45S and 5S rDNA sequences in *Paphiopedilum* subgenus *Parvisepalum* were consistently localized in sub-telomeric positions although the number and strength of signals varied between species (Figs. 1 and 3, Table 2). All species had at least one pair of chromosomes with co-localized 45S + 5S rDNA sites (see ideograms in Fig. 4). In *P. micranthum* and *P. delenatii* (Figs. 1e and f), no further 45S or 5S rDNA sites were detected, whereas *P. armeniacum*, *P. emersonii* and *P. hangianum* (Figs. 1a, c and d) had an additional chromosome pair with 45S rDNA signals in sub-telomeric positions. The most distinctive pattern was observed in *P. vietnamense* (Fig. 1g); in addition to the pair of chromosomes with co-localised 45S + 5S rDNA sites, three chromosome pairs had 45S rDNA sites in sub-telomeric regions. Two of these pairs of 45S rDNA signals were of much higher intensity and located on large chromosomes in contrast to the 45S rDNA signals of other species which were all located on smaller chromosomes and were much less intense.

**Table 2:** SatA and rDNA signals in seven species of *Paphiopedilum* subgenus *Parvisepalum*

Taxon	2n	Number of SatA sites				Number of rDNA sites		
		metacentric	sub-telocentric	interstitial	dispersed	45S	5S	45S + 5S co-localization
*P. armeniacum*	26	24	30			4	2	2
*P. delenatii*	26	6	14			2	2	2
*P. malipoense*	26	7	44		present	2	2	2
*P. micranthum*	26	6	16	14	present	2	2	2
*P. emersonii*	26	16				4	2	2
*P. hangianum*	26	14				4	2	2
*P. vietnamense*	28	2	14	2		8	4	2

## Discussion

### Global satellite repeat composition in *Paphiopedilum*

The analysis of satellite DNAs across an ever-increasing diversity of plants has shown that there is considerable diversity in sequence composition, diversity, organization, abundance and evolution [4, 6, 15]. Here we have taken advantage of high-throughput sequencing approaches and the bioinformatic pipeline RepeatExplorer to provide insights into the most abundant satellite DNA repeats (i.e. comprising ≥0.01% of the genome) across the seven subgenera/sections of the slipper orchid genus *Paphiopedilum*. As with some plant genomes analysed to date, e.g. *Silene latifolia* [50], the diversity of satellites identified was low, with only four major satellite types identified. Nevertheless, this contrasts with other plant genomes where a considerably greater diversity has been reported. For example, 51 different satellites were identified in *Vicia peregrina* [15] and up to 54 types, each comprising > 0.01% of the genome, were identified in *Luzula elegans* [45, 51].

Many plant satellites have been shown to be species-specific (e.g.in Fabeae, [15]), indicative of relatively rapid sequence divergence rates compared with speciation rate. However, this was not the case in *Paphiopedilum*, in which all four satellites were observed to be more widely distributed, either between more than one species in a subgenus (SatA) or between sections (SatJ), subgenera (SatB) or genera (SatG). The contribution of these four satellites to the whole genome varied considerably between species analysed, from just 0.13% of the genome in *P. concolor* (subgenus *Brachypetalum*) to 14.39% in *P. armeniacum* (subgenus *Parvisepalum*) (Table 1). In addition, there was no evidence of a clear correlation between satellite abundance and genome size. For example, both *P. concolor* (23.1 pg/1C) and *P. armeniacum* (22.8 pg/1C) have similar genome sizes, but the lowest and highest percentage of the genome comprising satellite DNAs of the species investigated (Table 1 and Additional file 2: Table S1). Such a situation has been observed in similar analyses in other groups [14, 15] in which it is generally transposable elements, particularly retrotransposons such as certain Ty1/Copia and Ty3/Gypsy elements, which correlate most closely with genome size [5, 15, 52, 53].

### Analysis of SatA: The most abundant satellite in *Paphiopedilum*

Plant genomes are often dominated by just one or a few repeats which have amplified to high copy number. Typically the most abundant repeats are retrotransposons. For example, the Ty3/Gypsy Ogre elements have been shown to comprise 54% of the genome of *Vicia faba* [15], whereas Ty3/Gypsy Gorge3 elements contribute up to 34% of the *Gossypium exiguum* genome [52]. Nevertheless, there are also examples in which satellites have contributed to a substantial proportion of the genome. For example, the VicTR-B satellite repeat represents about 25% of the *Vicia sativa* genome [54], whereas the FriSAT1 repeat accounts for up to 11% of the *Fritillaria affinis* genome [14], and the PaB6 repeat represents about 10% of the *Prospero autumnale* cytotype B^6^B^6^ genome [55]. In *Paphiopedilum*, the most abundant satellite DNA is SatA (Table 1) which is estimated to comprise c.14.39% of the *P. armeniacum* genome and is specific to subgenus *Parvisepalum* (Figs.1 and 2).

### Origin, evolution and organization of SatA in subgenus *Parvisepalum*

Characterization of SatA in *Paphiopedilum* subgenus *Parvisepalum* revealed just four (or conservatively two if CL_940, 393 and 886 are considered to be minor variants of a single sequence) primary monomers which have no homology to any satellite DNA sequences identified in other subgenera or sections of *Paphiopedilum*, the sister genus *Phragmipedium* or other plant genomes published to date. However, given that satellite DNA sequences are known to diverge rapidly and that subgenus *Parvisepalum* may have diverged from the rest of *Paphiopedilum* only c. 20 Mya [28], it is likely that SatA clusters arose independently in the lineage leading to subgenus *Parvisepalum*. The arrangement of the SatA and rDNA sites in *P. vietnamense* is highly distinctive in comparison with the other species. If *P. vietnamense* is sister to the rest of the subgenus, as indicated by ITS sequence divergence, then the distribution patterns in this species maybe either an apomorphic or plesiomorphic character state, or indeed a mixture of both character states, depending on the specific chromosomal signal.

Since the origin of SatA, there have clearly been considerable changes in its abundance and chromosomal distribution between the different species comprising subgenus *Parvisepalum*. However, despite this variability, most signals occur at peri-centromeric or sub-telocentric regions of the chromosome, as is typical of many satellites [22]. When the distribution patterns are considered in a phylogenetic context (Figs. 3 and 4), it is apparent that the most closely related species carry the most similar satellite DNA distributions and that the distribution of satellites carries a strong phylogenetic signal.

The plethora of hemizygous interstitial sites in *P. micranthum* indicates rapid divergence of SatA within a species, perhaps associated with genetic drift in local populations and some gene flow between populations. Further studies of *P. micranthum* at the population level are clearly needed to provide deeper insights into satellite DNA proliferation in this species.

### Organization of rDNA sites in subgenus *Parvisepalum*

Previous studies of the organization of rDNA sequences in *Paphiopedilum* have suggested that the ancestral state for the genus is two 45S and two 5S rDNA sites [38]. Although the number of 5S rDNA sites was observed to be maintained throughout subgenus *Parvisepalum*, in our study we found that the number of 45S rDNA sites varied from one to eight (Table 2, Fig. 4), in contrast to the range from two to four previously reported [38]. Indeed, the *P. delenatii*, *P. micranthum* and *P. malipoense* 5S and 45S rDNA FISH profiles in this study (Figs. 1b, e and f, Table 2) differed from those of Lan and Albert [38], which suggests that there may well be intraspecific rDNA site number variation in these species.

### Ancestral chromosome number of *Paphiopedilum*

The new karyotype of *P. vietnamense*, with 2n = 28, metacentric chromosomes reported here (Figs. 3 and 4), expands the chromosome number range for subgenus *Parvisepalum*, which was previously considered to be conserved at 2n = 26. Prior to this study, 2n = 28 was only reported for *P. hookerae* and *P. sangii* which both belong to section *Barbata* [36, 56]. Previously it was suggested that *Paphiopedilum* had an ancestral chromosome number of 2n = 26 comprising metacentric chromosomes [57]. However, confidence in this number is dependent on the precise placement of *P. vietnamense* in phylogenetic trees for the genus. If *P. vietnamense* is sister to the rest of subgenus *Parvisepalum,* as suggested from the nrITS sequence data (Additional file 1: Fig. S1), then the ancestral chromosome number of subgenus *Parvisepalum* could be 2n = 26 or 28. However, if *P. armeniacum* is sister to the rest of the subgenus, as suggested by plastid DNA sequences [28], then 2n = 26 is the most likely ancestral chromosome number of the subgenus. Given that 2n = 26 or 2n = 28 are both possible ancestral chromosome numbers for subgenus *Parvisepalum,* and because subgenus *Parvisepalum* is sister to the rest of *Paphiopedilum* [28], the ancestral chromosome number of the genus itself could also be 2n = 26 or 2n = 28. Chromosome numbers from other genera in Cypripedioideae do not support one the alternatives above the other, although perhaps they point most strongly towards an ancestral chromosome number of 2n = 26 for Cypripedioideae. This is because the sister genera *Phragmipedium* and *Mexipedium* are together sister to *Paphiopedilum* and they have chromosome numbers of 2n = 18–30 for *Phragmipedium* and 2n = 26 for *Mexipedium* (a monotypic genus) (Chromosome counts database, http://ccdb.tau.ac.il/search/Phragmipedium/). However, we do not currently know how the range of chromosome numbers of *Phragmipedium* species are distributed across the phylogenetic tree for the genus and without that we cannot readily determine the ancestral number of *Phragmipedium* and *Mexipedium*. Thus, in determining the chromosome ancestry of *Paphiopedilum*, we need greater clarity of the phylogenetic placement of *P. vietnamense* and of the evolution of chromosome numbers in *Phragmipedium*.

## Conclusion

We identified and characterized a new satellite repeat, SatA from *P. armeniacum* using the RepeatExplorer pipeline to analyse HiSeq Illumina reads. SatA is specific to subgenus *Parvisepalum* but absent from the other two subgenera in *Paphiopedilum*. Since the distribution pattern of SatA on chromosomes in subgenus *Parvisepalum* is species-specific and hence rapidly evolving, and possesses a strong phylogenetic signal, it is an ideal probe that could be used as a chromosomal marker for characterizing karyotype evolution in species belonging to subgenus *Parvisepalum*. Nevertheless, greater certainty is needed on the phylogenetic placement of *P. vietnamense*, as this will help to shed light on the ancestral karyotype and chromosome number of the genus. Certainly, it is recognized that the currently available phylogenetic data for *Paphiopedilum* is limited and could be greatly enhanced by the application of phylogenomic and bioinformatic approaches, involving multiple genes, repeats or even whole genomes, to generate robust species trees and phylogenetic insights of the genus.

## Methods

### Plant materials

Details of the origin of plant materials used for Illumina sequencing, fluorescent in situ hybridization (FISH), and genome size estimations and the location of the voucher specimens are given in Additional file 7: Table S3. All other nrITS sequences used in the phylogenetic analysis were downloaded from GenBank.

### Genome size estimation using flow cytometry

Flow cytometry (FCM) was used to determine the genome sizes of each studied species and estimate the volume of Illumina sequence DNA required to characterize the repetitive fraction of the genome (see below). Samples for FCM were prepared as in Ebihara et al. [58] with slight modifications. Briefly, c. 1 cm^2^ of leaf material of the *Paphiopedilum* sample was co-chopped with the reference standard *Vicia faba* ‘Inovec’ (2C = 26.9 pg, [59]) in 2.0 mL of Ebihara buffer [1.0% Triton X-100, 140 mM 2-mercaptoethanol, 50 mM Na_2_SO_3_, 50 mM Tris-HCl (pH 7.5), 40 mg/mL polyvinyl-pyrolidone (PVP-40) and 0.1 mg/mL ribonuclease] on ice in a fume hood. The resulting slurry was incubated for 5 min on ice and filtered through a 30-μm nylon mesh (Partec) into a 2.0-mL tube and the nuclei were subsequently stained with 100 μL propidium iodide (1 mg/mL). The filtrate was incubated for 15 min at 37 °C and then left on ice for c. 30 min. Genome sizes were measured on a PartecCyflow SL3 flow cytometer (Partec GmbH, Münster, Germany) fitted with a 100 mW green solid state laser (532 nm, Cobolt Samba, Solna, Sweden). As many orchids, *Paphiopedilum* presents endoreplication [60] and we had to run the samples longer than usual to recover 1000 nuclei in the 2C peak of G1-phase for *Paphiopedilum*. Three measurements were made for each sample. The output histograms were analysed with the FlowMax software v.2.4 (Partec GmbH).

### High-throughput sequencing of genomic DNA

Total genomic DNA was isolated from young leaf samples using DNeasy Plant Mini Kit (Qiagen, Hilden, Germany) according to the manufacturer’s instructions. Paired-end sequencing (2× 100 bp or 2 × 125 bp, 300–500 bp insert size) of total genomic DNA was performed using Illumina HiSeq 2000 (Illumina, Inc.) at Genomics BioSci & Tech. Co., Ltd., Taipei, Taiwan and using a HiSeq 2500 at BGI, Hong Kong and Service XS, Leiden, The Netherlands, based on libraries made by the service providers.

### Processing of Illumina HiSeq data

The quality of the raw reads was assessed by Fast QC v0.10.1. Raw reads were trimmed and quality-filtered with a FASTX-Toolkit v 0.013 to give 90-bp length reads with 90% of bases having a minimum Phred score of 20. To remove sequences of organellar origin, custom Perl scripts and the stand-alone version of BLAST v2.2.16 [60] were used to screen the quality-filtered reads against a custom database containing the draft plastid genome of *Phragmipedium longifolium* (unpublished data provided by Dr. W. Mark Whitten, University of Florida, USA) and the published mitochondrial genomes of ten monocots (taken from GenBank). Parameter settings used for BLASTN [61] searches were: -v 1 -G 0 -E 2 -K 0 -b 0 - e 0.000001 -F mL. Sequences with similarity matches to the database (*E*-value ≤19 × 10^− 6^) were removed. All remaining reads were considered to be of nuclear origin and uploaded to RepeatExplorer (http://www.repeatexplorer.org) within the Galaxy server environment, as described in Dodsworth et al. [62]. Nuclear reads were paired using the FASTQ interlacer tool implemented in RepeatExplorer [63, 64]. Overlapping read pairs, which are caused by the presence of overly short genomic DNA fragments in the sequence library, can adversely affect the subsequent RepeatExplorer clustering analysis. Consequently, they were removed using a RepeatExplorer utility (minimum overlap = 30 nt, maximum mismatch per 100 bp = 1, offset = 5).

### Clustering and annotation of satellite DNA with RepeatExplorer (RE)

The RepeatExplorer pipeline runs a graph-based clustering algorithm [63, 64] to assemble the groups of frequently overlapping reads into clusters of reads, each representing a repetitive element, or part of a repetitive element with a higher order genome structure. Similarity- and structure-based repeat identification tools in RepeatExplorer aid the identification of the repeats. RepeatExplorer uses a BLAST threshold of 90% similarity across 55% of the read to identify reads to each clusters (minimum overlap = 55, cluster threshold = 0.01%, minimum overlap for assembly = 40), and the clusters are identified based on a principle of maximum modularity.

To identify major shared and unique repetitive elements between *Paphiopedilum* species, comparative clustering analysis was performed on a combined dataset comprising Illumina sequence reads from seven *Paphiopedilum* species representing each of the seven major *Paphiopedilum* subgroups, and *Phragmipedium* as an outgroup (see Additional file 2: Table S1). For each taxon, the number of HiSeq Illumina reads analysed in RepeatExplorer was scaled according to the genome size of the species, so that the same proportion of the genome (2%) was analysed in each case. To provide greater insights into the characteristics of individual repetitive elements identified in repeat clusters, these eight HiSeq samples were also analysed in RepeatExplorer individually. For that we used the highest possible number of reads that can be readily handled by the software (Additional file 2: Table S1). Preliminary runs of RepeatExplorer indicated that the *P. armeniacum* sample contained a high (> 10%) proportion of satellite DNA. Large repeat clusters containing many sequence reads, and identified as comprising tandem repeats (e.g. satellites) can be problematic in RepeatExplorer analysis as they consume high amounts of computing resources and so reduce the number of sequences that can be handled by the pipeline. Consequently, prior to clustering, the reads were subjected to a custom sequence filter (containing a database of 617 of the most abundant sequence contigs from the satellite SatA, see below) to remove 90% of the satellite sequences, as recommended by the developers of RepeatExplorer. Clusters were identified manually as described in Novák et al. [64] by scanning read similarity hits to the RepeatMasker [65] database, visual examination of graphs and the location of cluster mates. Only clusters with genome proportions (GP) > 0.01% were included in subsequent analyses.

### Characterization of the most abundant satellite repeat in *Paphiopedilum*

The structural features of the top four monomers of the most abundant satellite repeat identified by RepeatExplorer (SatA) were characterized using DOTTER [66] and the Vienna RNA fold tool implemented in Geneious v. 9.0.5 [67]. Sequence homology was checked against other *Paphiopedilum* satellites and by BLAST against published satellite DNA sequences in PlantSat (http://w3lamc.umbr.cas.cz/PlantSat/) [68].

### PCR amplification, cloning and sequencing of SatA

The consensus sequence of the SatA monomer was used to design oligonucleotide primers (CL3C37-F:CATTTTCAACGTCGAGCC; CL3C37-R:AGACAAATTCTAAGCTATATGGAC; PCR product 147 bp) for amplifying the SatA monomers from genomic DNA of *P. armeniacum*. The amplification was performed in a reaction volume of 20 μl using the KAPA HiFi HotStart PCR kit (KAPA Biosystems) in a Takara thermal cycler (Takara Bio, Shiga, Japan), and the reaction mixture contained 1× KAPA HiFi buffer, 0.3 mM each dNTP, 0.3 μM each primer, and 1unit KAPA HiFi HotStart DNA polymerase. The amplification profile included an initial step at 94 °C for 3 min, followed by 35 cycles of 94 °C for 15 s, 50 °C for 15 s, and 72 °C for 5 s, and finally a 1 min final extension at 72 °C. DNA was cloned using the T&A cloning vector system (Yeastern Biotech Co., Ltd., Taiwan) and DH5α competent cells (Genomics BioSci & Tech. Co., Ltd., Taiwan) following the manufacturer’s instructions.

### Chromosome preparation

Chromosome preparations for FISH were made according to the methods of Chung et al. [69] with minor modifications. Briefly, young, healthy root tips were harvested and pretreated in 2 mM 8-hydroxyquinoline at 18 °C for 5 h to accumulate metaphase nuclei, rinsed with distilled water and then fixed in freshly prepared Farmer’s fluid (3:1 ethanol:glacial acetic acid). Root tips were macerated with 6% cellulose (Onozuka R-10, Yakult Honsha, Japan) and 6% pectinase (Sigma Chemical Co., St. Louis, Mo.) in 75 mM KCl, pH = 4.0 at 37 °C for 90 min, and squashed on a microscope slide in the same fixative. Slides were air-dried and stored at − 80 °C until required.

### Fluorescent in situ hybridization

The FISH procedure followed that described in Lee et al. [70]. The SatA probe was designed from a conserved region of the SatA monomer alignment and labeled with digoxigenin using oligonucleotide-5′-end-labeling (AAATCTGACCTAATTTGGACCCAATCTTTGAACCTTCTAATTGAAGGTCAATTGGTGT). Probes for 45S rDNA (pTA71 containing a repetitive unit of 45S rDNA from *Triticum aestivum*) [71] and 5S rDNA (pTA794 containing the 5S rDNA repeat unit from *T. aestivum*) [72] were also used. The rDNA sequences were labeled by nick translation with digoxigenin-11-dUTP orbiotin-16-dUTP (Roche Diagnostics GmbH, Penzberg, Germany). Digoxigenin-labeled probes were detected by anti-digoxigenin-rhodamine (Roche Diagnostics GmbH), whereas biotin-labeled probes were detected using fluorescein isothiocyanate (FITC)-conjugated avidin (Vector Laboratories, Burlingame**,** CA, USA). Chromosomes were counterstained with 4′, 6-diamidino-2-phenylindole (DAPI) in an anti-fade solution (Vector Laboratories, CA, USA). All images were captured digitally using a CCD camera attached to an epifluorescence microscope (Axioskop 2, Carl Zeiss AG, Germany). The CCD camera was controlled by Image-Pro Plus software (version 4.5.1, Media Cybernetics, Yorktown, VA, USA), and final image adjustments were made with Adobe Photoshop CS2 (version 9.0.2, Adobe Systems Inc., San Jose, CA, USA).

### Analysis of phylogenetic relationships in *Paphiopedilum*

DNA was extracted from each sample using a DNeasy Plant Mini Kit (Qiagen, Hilden, Germany). The nrITS regions, which include ITS1, ITS2 and the 5.8S nuclear rRNA gene, were amplified by PCR with the primer combinations of Sun et al. [73] and White et al. [74] and Sanger sequenced as described in Chochai et al. [27]. PCR products of *P. emersonii* were difficult to sequence directly and thus were cloned using the pGEM-T Vector System II (Promega, Madison, WI, USA). This yielded five distinct nrITS sequences which were verified using a BLAST search against the NCBI sequence database (National Center for Biotechnology Information, GenBank). GenBank accession numbers (http://www.ncbi.nlm.nih.gov) of nrITS sequences were listed in Additional file 8: Table S4.

Phylogenetic relationships between the seven *Paphiopedilum* species comprising subgenus *Parvisepalum* were analysed using the newly generated nrITS sequences together with those downloaded from GenBank for representative species of the other subgenera/sections of *Paphiopedilum* and with *Phragmipedium besseae* and *P. longifolium* as outgroup species. Sequences were aligned using CLUSTALW [75] implemented in Geneious v.9.0.5 and checked by eye. Phylogenetic relationships were analysed using a model-based Bayesian approach with MrBayes 3.2.1 [76]. The ‘best-fit’ model of evolution was selected under the Akaike information criterion test [77] as implemented in MrModel test 2.2 [78]. The general time reversal plus invariant rates and a gamma distribution (GTR + I + Γ) model was selected for the analyses. Two separate runs of four Monte Carlo Markov chains (MCMC; Yang and Rannala [79]) were performed for 10,000,000 generations until the mean deviation of split frequency dropped below 0.01, and a tree was sampled every 1000th generation. Trees from the first 25% of generations were discarded using the “burn-in” command, and the remaining trees were used to calculate an all-compatible consensus topology and posterior probability (PP) values for individual branches. The alignment datasets were further analysed using maximum parsimony (MP) in PAUP* version 4.0b10 [80]. Support for groups was evaluated using the bootstrap method [81] with 1000 replicates. The trees obtained in these analyses were drawn with the TreeGraph 2 software [82].

## Additional files

Additional file 1: **Figure S1.** A MrBayes ITS subtree showing relationships in *Paphiopedilum* subgenus *Parvisepalum* is presented. Numbers above branches indicate bootstrap and posterior probability support values. Length of branches indicate number of changes. Numbers on tips indicate ITS clones. (JPG 186 kb)

Additional file 2: **Table S1.** Reads clustered and genome proportion for RepeatExplorer (RE) analysis. (XLSX 12 kb)

Additional file 3: **Table S2.** Characteristics of the top four-most SatA abundant monomers. (DOCX 12 kb)

Additional file 4: **Figure S2.** Hypothetical folding of the four most abundant SatA monomers: (A) CL1_965, (B) CL1_940, (C) CL1_393 and (D) CL1_886, when viewed as continuous molecules following the DNA energy model (Mathews 2004) implemented in Geneious v9.0.5. The repeat/inverted repeats in the monomers pair and fold to form hairpin-loop structures. (JPG 209 kb)

Additional file 5: **Figure S3.** Dot plots for the four most abundant SatA monomers: (A) CL1_965, (B) CL1_940, (C) CL1_393 and (D) CL1_886, by DOTTER2 (Sonnhammer and Durbin 1995) implemented in Geneious v 9.0.5. (JPG 539 kb)

Additional file 6: **Figure S4.** Sequence of the most abundant SatA monomers: (1) CL1_965, (2) CL_940, (3) CL1_393 and (4) CL1_886. Annotations show positions of the major subunits and major (> 10 bp long) repeat/inverted regions. (JPG 704 kb)

Additional file 7: **Table S3.** The vouchers and sources used for Illumina HiSeq, RepeatExplorer (RE) clustering, FISH and genome size estimation in this study. (DOCX 18 kb)

Additional file 8: **Table S4.** GenBank accession number of nrITS sequences used in phylogenetic analysis. (XLSX 14 kb)

## Abbreviations

CCD: Charge-coupled device
DAPI: 4′, 6-diamidino-2-phenylindole
FCM: Flow cytometry
FISH: Fluorescent in situ hybridization
FITC: Fluorescein isothiocyanate
GP: Genome proportions
MCMC: Monte Carlo Markov chains
nrITS: Nuclear ribosomal internal transcribed spacer region
PCR: Polymerase chain reaction
PP: Posterior probability
PVP: Polyvinyl-pyrolidone
rDNA: Ribosomal DNA
RE: RepeatExplorer
SAT: Satellite DNA
