# Supplementary figures and images for: Satellite DNA in Paphiopedilum subgenus Parvisepalum as revealed by high-throughput sequencing and fluorescent in situ hybridization
Source: BMC Genomics. 2018 Aug 2;19:578. doi: 10.1186/s12864-018-4956-7 (PMC6090851; doi:10.1186/s12864-018-4956-7)

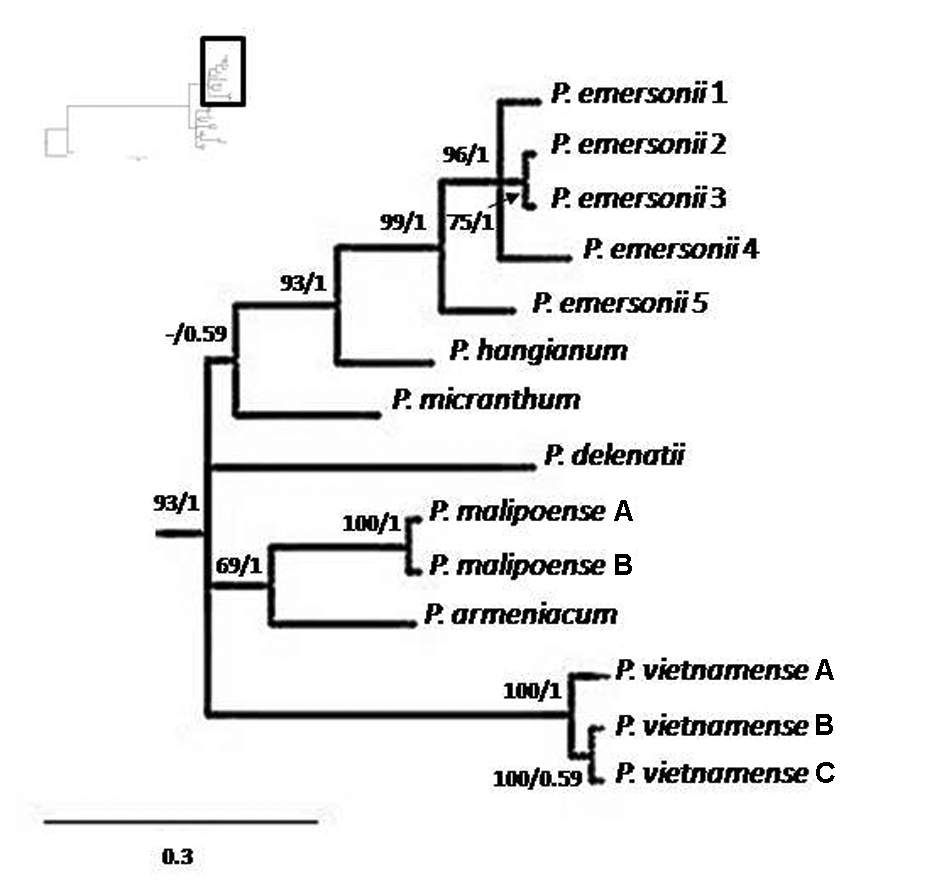

Supplement: Supplementary file 1 — Figure S1. A MrBayes ITS subtree showing relationships in Paphiopedilum subgenus Parvisepalum is presented. Numbers above branches indicate bootstrap and posterior probability support values. Length of branches indicate number of changes. Numbers on tips indicate ITS clones. (JPG 186 kb) [file 12864_2018_4956_MOESM1_ESM.jpg]

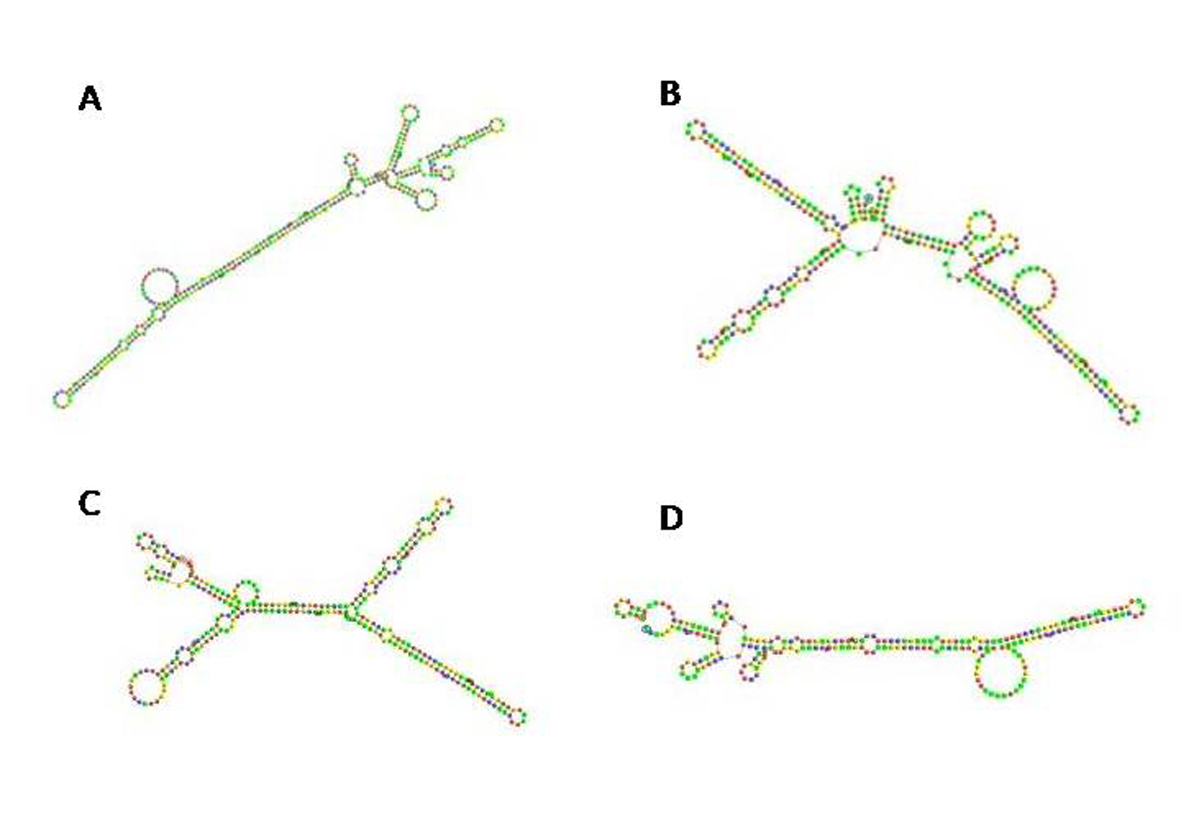

Supplement: Supplementary file 4 — Figure S2. Hypothetical folding of the four most abundant SatA monomers: (A) CL1_965, (B) CL1_940, (C) CL1_393 and (D) CL1_886, when viewed as continuous molecules following the DNA energy model (Mathews 2004) implemented in Geneious v9.0.5. The repeat/inverted repeats in the monomers pair and fold to form hairpin-loop structures. (JPG 209 kb) [file 12864_2018_4956_MOESM4_ESM.jpg]

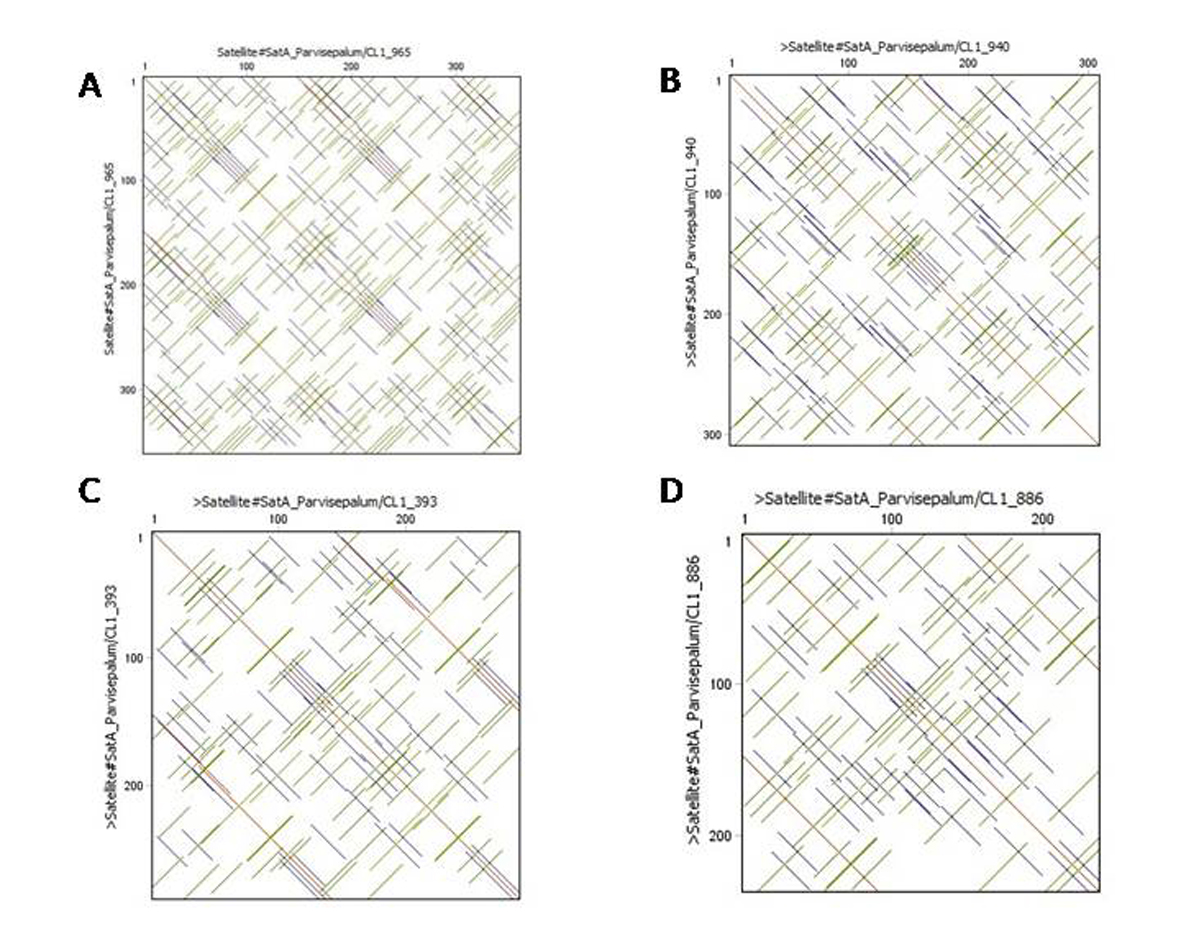

Supplement: Supplementary file 5 — Figure S3. Dot plots for the four most abundant SatA monomers: (A) CL1_965, (B) CL1_940, (C) CL1_393 and (D) CL1_886, by DOTTER2 (Sonnhammer and Durbin 1995) implemented in Geneious v 9.0.5. (JPG 539 kb) [file 12864_2018_4956_MOESM5_ESM.jpg]

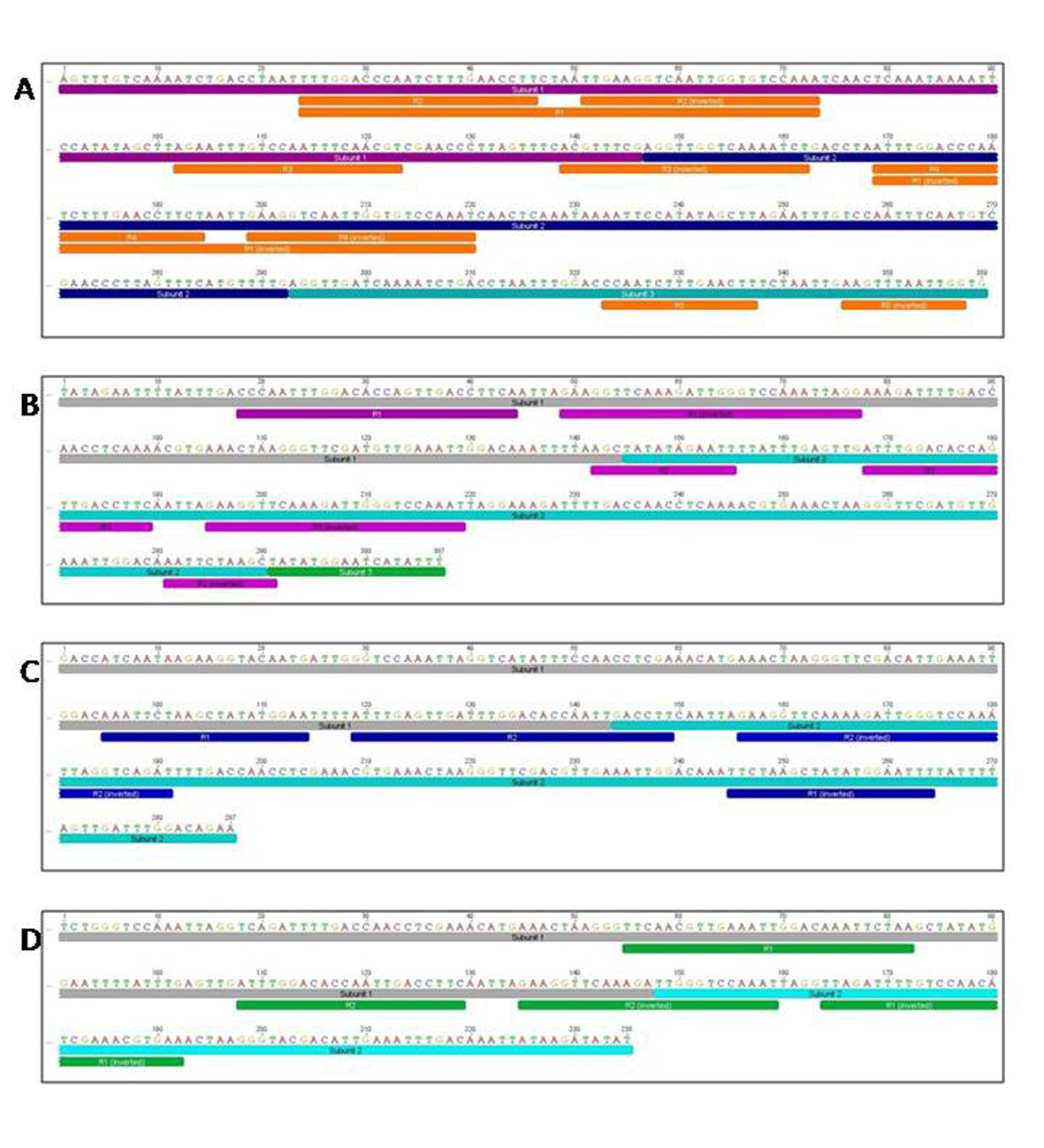

Supplement: Supplementary file 6 — Figure S4. Sequence of the most abundant SatA monomers: (1) CL1_965, (2) CL_940, (3) CL1_393 and (4) CL1_886. Annotations show positions of the major subunits and major (> 10 bp long) repeat/inverted regions. (JPG 704 kb) [file 12864_2018_4956_MOESM6_ESM.jpg]
