# Supplementary material for: Satellite DNA in Paphiopedilum subgenus Parvisepalum as revealed by high-throughput sequencing and fluorescent in situ hybridization
Source: BMC Genomics. 2018 Aug 2;19:578. doi: 10.1186/s12864-018-4956-7 (PMC6090851; doi:10.1186/s12864-018-4956-7)
Supplement: Supplementary file 3 — Table S2. Characteristics of the top four-most SatA abundant monomers. (DOCX 12 kb) [file 12864_2018_4956_MOESM3_ESM.docx]

**Additional file 2: Table S1** Characteristics of the top five-most SatA abundant monomers.

| Monomer contig | Length (bp) | A+T content (%) | Similarity hits (%) |
| --- | --- | --- | --- |
| CL1_965 | 359 | 65.7 | 49.8 |
| CL1_940 | 307 | 66.4 | 20.9 |
| CL1_393 | 287 | 65.2 | 10.7 |
| CL1_886 | 235 | 66.0 | 10.4 |
