# Supplementary material for: Satellite DNA in Paphiopedilum subgenus Parvisepalum as revealed by high-throughput sequencing and fluorescent in situ hybridization
Source: BMC Genomics. 2018 Aug 2;19:578. doi: 10.1186/s12864-018-4956-7 (PMC6090851; doi:10.1186/s12864-018-4956-7)
Supplement: Supplementary file 7 — Table S3. The vouchers and sources used for Illumina HiSeq, RepeatExplorer (RE) clustering, FISH and genome size estimation in this study. (DOCX 18 kb) [file 12864_2018_4956_MOESM7_ESM.docx]

**Additional file 6: Table S2** Information on plant vouchers and sources used for Illumina HiSeq, RepeatExplorer (RE) clustering, FISH and genome size estimation in this study.

| Taxon ^a^ | Vouchers for RepeatExplorer and phylogenetic analyses | Vouchers for FISH experiments | Vouchers for genome size estimation |
| --- | --- | --- | --- |
| **Subgenus *Parvisepalum*** |  |  |  |
| *P. armeniacum* | Yung-I Lee 201101 (TNM) | Yung-I Lee 201101 (TNM) | X-0779 (MBG) |
| *P. delenatii* | -- | Yung-I Lee 201001 (TNM) | Yung-I Lee 201001 (TNM) |
| *P. malipoense* | -- | Yung-I Lee 201002 (TNM) | Yung-I Lee 201002 (TNM) |
| *P. micranthum* | -- | Yung-I Lee 201003 (TNM) | Yung-I Lee 201003 (TNM) |
| *P. emersonii* | -- | Yung-I Lee 201103 (TNM) | Yung-I Lee 201103 (TNM) |
| *P. hangianum* | -- | Yung-I Lee 201104 (TNM) | Yung-I Lee 201104 (TNM) |
| *P. vietnamense* | -- | Yung-I Lee201109 (TNM) | Yung-I Lee201109 (TNM) |
| **Subgenus *Brachypetalum*** |  |  |  |
| *P. concolor* | Yung-I Lee 201201 (TNM) | Yung-I Lee 201201 (TNM) | HBL 20110263 (HBL) |
| **Subgenus *Paphiopedilum*** |  |  |  |
| **Section *Paphiopedilum*** |  |  |  |
| *P. druryi* | HBL 2013 0012 (HBL) | -- | -- |
| *P. fairrieanum* | HBL 20110265 (HBL) | -- | -- |
| *P. henryanum* | HBL 20110255 (HBL) | -- | -- |
| *P. villosum* | Yung-I Lee 201012 (TNM) | Yung-I Lee 201012 (TNM) | K20052739 (K); K20101520 (K) |
| **Section *Coryopedilum*** |  |  |  |
| *P. glanduliferum* A | HBL 1071 (HBL) | -- | -- |
| *P. glanduliferum* B | KAS 21 1071 (HBL) | -- | -- |
| *P. rothschildianum* | Yung-I Lee 201107 (TNM) | Yung-I Lee 201107 (TNM) | K19844033/36806 (K) |
| **Section *Pardalopetalum*** |  |  |  |
| *P. lowii* | HBL 30629 (HBL) | Yung-I Lee201356 (TNM) | HBL 30629 (HBL) |
| **Section *Barbata*** |  |  |  |
| *P. appletoniannum* | Yung-I Lee 201209 (TNM) | Yung-I Lee 201209 (TNM) | QM3020 (QM); QM3023 (QM) |
| *P. barbatum* | HBL 200070142 (HBL) | -- | -- |
| *P. purpuratum* | HBL 20110252 (HBL) | -- | -- |
| **Section *Cochlopetalum*** |  |  |  |
| *P. primulinum* | K19811628 (K) | Yung-I Lee 201115(TNM) | K19811628 (K) |
| **Outgroup** |  |  |  |
| *Phragmipedium longifolium* | HBL 20110235 (HBL) | -- | HBL 20110235 (HBL) |

^a^The taxonomic system of Cribb (1998) is followed.

HBL: Hortus botanicus of Leiden University, The Netherlands

K: Royal Botanic Gardens, Kew, UK

MBG: Munich Botanic Gardens, Germany

QM: Queen Mary University of London, UK

TNM: National Museum of Natural Science, Taiwan
